# Supplementary figures and images for: Transcriptome Profiling of a Soybean Mutant with Salt Tolerance Induced by Gamma-ray Irradiation
Source: Plants (Basel). 2024 Jan 16;13(2):254. doi: 10.3390/plants13020254 (PMC10818854; doi:10.3390/plants13020254)

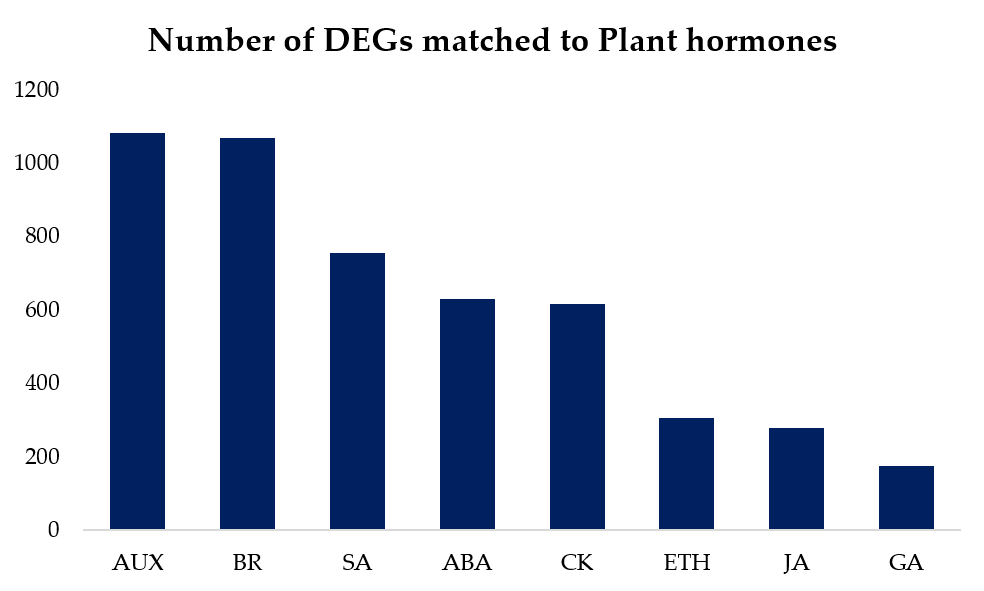

Supplement: Supplementary file 1 [file plants-13-00254-s001.zip › Figure S1.tif]

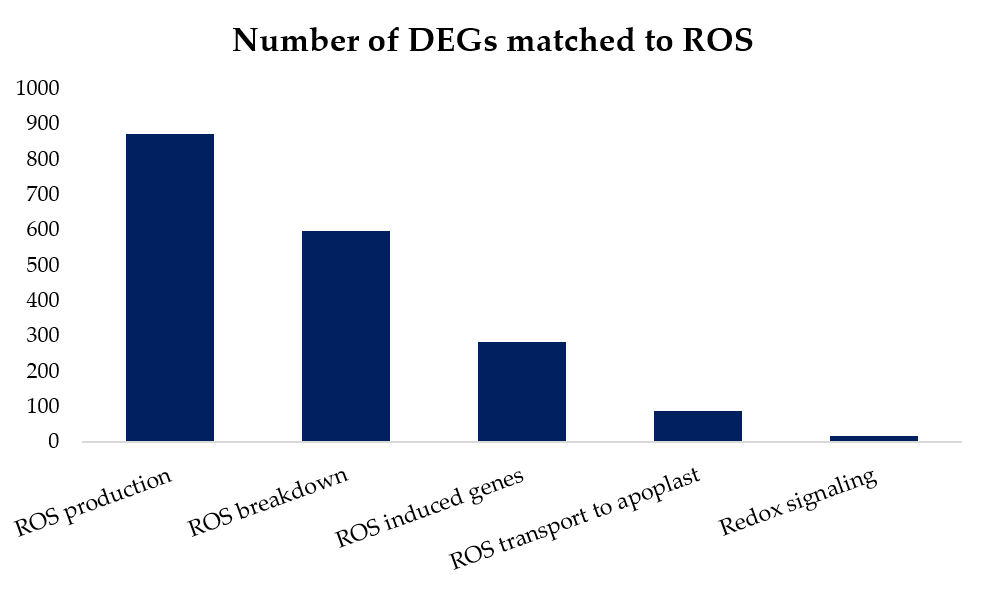

Supplement: Supplementary file 1 [file plants-13-00254-s001.zip › Figure S2.tif]

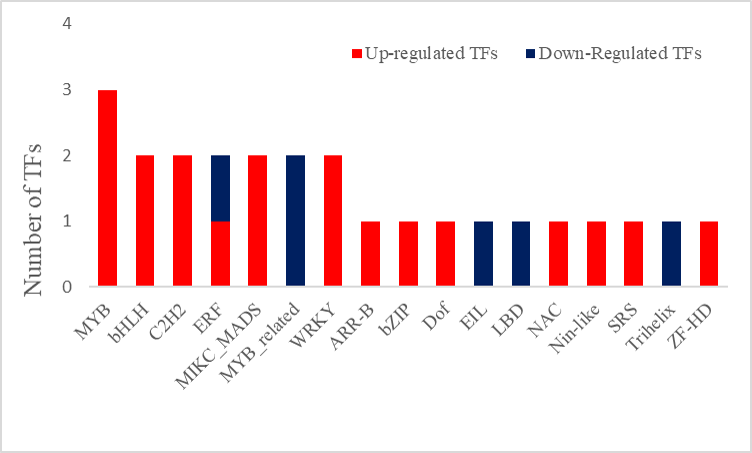

Supplement: Supplementary file 1 [file plants-13-00254-s001.zip › Figure S3.tif]
